# Elasticity of Semiflexible ZigZag Nanosprings with a Point Magnetic Moment

**DOI:** 10.3390/polym15010044

**Published:** 2022-12-22

**Authors:** Mohammadhosein Razbin, Panayotis Benetatos

**Affiliations:** 1 Department of Energy Engineering and Physics, Amirkabir University of Technology, Tehran 14588, Iran; 2 Department of Physics, Kyungpook National University, 80 Daehakro, Bukgu, Daegu 41566, Republic of Korea

**Keywords:** wormlike chain, kinked polymers, nanosprings, magnetometry, actuators

## Abstract

Kinks can appear along the contour of semiflexible polymers (biopolymers or synthetic ones), and they affect their elasticity and function. A regular sequence of alternating kink defects can form a semiflexible nanospring. In this article, we theoretically analyze the elastic behavior of such a nanospring with a point magnetic dipole attached to one end while the other end is assumed to be grafted to a rigid substrate. The rod-like segments of the nanospring are treated as weakly bending wormlike chains, and the propagator (Green’s function) method is used in order to calculate the conformational and elastic properties of this system. We analytically calculate the distribution of orientational and positional fluctuations of the free end, the force-extension relation, as well as the compressional force that such a spring can exert on a planar wall. Our results show how the magnetic interaction affects the elasticity of the semiflexible nanospring. This sensitivity, which is based on the interplay of positional and orientational degrees of freedom, may prove useful in magnetometry or other applications.

## 1. Introduction

The wormlike chain (WLC) model is a widely accepted minimal theoretical model of semiflexible polymers [1,2,3]. It is a locally inextensible one-dimensional line with bending stiffness. Tuning the bending stiffness, which, in turn, determines the persistence length, $l_{p}$, this model can be applied to the entire spectrum between the two extreme cases of the random coil and the rigid rod. Many important biopolymers are reasonably represented as WLCs. For example, DNA, the structural elements of the cytoskeleton (F-Actin, microtubules, intermediate filaments), and collagen can be viewed as WLCs over a wide range of persistence lengths. In addition, several synthetic polymers can be modeled as semiflexible WLCs [4]. Single-walled carbon nanotubes have a persistence length (at T= 300 K) of about 0.03–1 mm [5,6]. DNA nanorods have a persistence length of the order of 1–5 μm [7,8]. Thermal fluctuations in accordance with the WLC model have also been observed in very stiff superparamagnetic microrods with $l_{p}$ of the order of tens of meters [9].

Kinks along the contour of a semiflexible polymer can appear, and they affect its elasticity and function. Cisplatin is a chemotherapy drug used to treat cancerous tumors. The attachment of a cisplatin molecule to the side of DNA induces a kink with a well-defined bending angle, which eventually leads to the death of cancerous cells [10]. In chromatin, the wrapping of DNA around a histone octamer can be viewed as effectively inducing a kink in the sequence of linker DNA [11]. Another example is the zigzag structure of regular kinks in the Sorona 3GT polymer, which is used in the textile industry [12]. Kinks can also be formed in carbon nanotubes. Pairs of disclinations (heptagon-pentagon) at diametrically opposite points along a single-walled carbon nanotube can produce a kink [13,14]. Their creation through bending requires a lot of energy, as it involves the breaking of covalent bonds. Kinks in single-walled and multiwalled carbon nanotubes can also be created through buckling of the high curvature region. These kinks are fully reversible, but in order to be maintained, they require the exertion of a finite bending moment [15]. An alternative way to produce stable kinks in multiwalled carbon nanotubes and in other filamentous structures, such as the acrosomal process of the *Limulus* sperm (a rod of bundled actin filaments), has been described by Cohen and Mahadevan and involves slipping of the outer fibers relative to the inner ones [16]. Kinks can also be created in DNA nanorods using the sub-nanometer precision techniques of DNA origami nanotechnology [17,18,19,20,21,22,23,24,25,26,27,28,29]. Yet another way to get a zigzag structure at the nanolevel could be the so-called chevron graphene nanoribbons [30]. These are extended (ribbon-like) structures, but if the arms are long and thin enough at a coarse-grained level, they can be viewed as two-dimensional semiflexible zigzag nanosprings.

A theoretical study by Razbin [31] analyzed the elasticity of a grafted two-dimensional nanospring consisting of weakly bending (rod-like) semiflexible polymers connected by a sequence of alternating kinks. The longitudinal compliance was found to be proportional to the number of segments, whereas the transverse compliance was proportional to the cube of the number of segments.

Magnetoelastic coupling in soft materials has become an area of intensive research activity in recent years because it can be exploited to actuate soft structures without any mechanical contact [32]. One way to realize this coupling is by embedding magnetizable colloidal particles into an elastic polymeric matrix [33]. At the single-molecule level, magnetic filaments consisting of magnetic or magnetizable beads linked together by short polymer segments have been studied both theoretically and experimentally [34,35,36,37,38,39], and have been used in applications, e.g., in order to construct an artificial flagellum [40]. In a recent publication, we theoretically analyzed the elastic response of a grafted semiflexible nunchuck (two weakly bending rod-like polymers linked together by a soft hinge) having a magnetic moment at the free end. We found significant sensitivity of the conformational fluctuations to the magnetic interaction, which could, in principle, be used in magnetometry [41]. The nonlinear elasticity of a grafted Euler beam with a magnetic bead at the free end has been analyzed in [42], albeit ignoring thermal fluctuations. Similar systems have also been studied using engineering modeling [43,44,45].

In this article, we analyze the elasticity of a grafted two-dimensional kinked filament structure with a magnetic bead at the end. The filament structure can be viewed as a model of a semiflexible nanospring. The nanospring is assumed to be grafted to a substrate while the free endpoint of the structure is attached to a point dipole moment. Subjecting the system to a uniform and constant magnetic field, we obtain analytic expressions for the probability density of the position and the orientation of the endpoint of the filament. This allows us to calculate the longitudinal and transverse force-extension relations of the filament. We also confine the system with a rigid impenetrable wall and calculate the force exerted by the endpoint of the structure on the wall. All of the calculations are based on the Gaussian approximation of the WLC model, which is valid in the weakly bending regime (the stiff chain limit). The Gaussian two-point positional-orientational probability (propagator or Green’s function) has been used in the theoretical analysis of various systems of rod-like elements, such as semiflexible nunchucks [41,46], branched actin filaments [47], and semiflexible quadrilaterals [48].

This article is organized as follows. In Section 2, we review the two-point positional and/or orientational probability density for a weakly bending wormlike chain. In Section 3, we consider a grafted filament with a regular sequence of alternating kinks (in two dimensions), without the magnetic bead and calculate the positional and orientational probabilities of the free end. In Section 4, we calculate the same conformational probabilities for a grafted kinked filament with a magnetic moment attached to the free end, interacting with a uniform magnetic field. In Section 5, we calculate the linear response (force-extension relation) of this system in the longitudinal and in transverse directions. In Section 6, we calculate the compressional force exerted by the fluctuating end of this system on a confining planar wall. We discuss our results and summarize them in Section 7.

## 2. Single-Grafted Weakly Bending Filament

**Figure 1:**
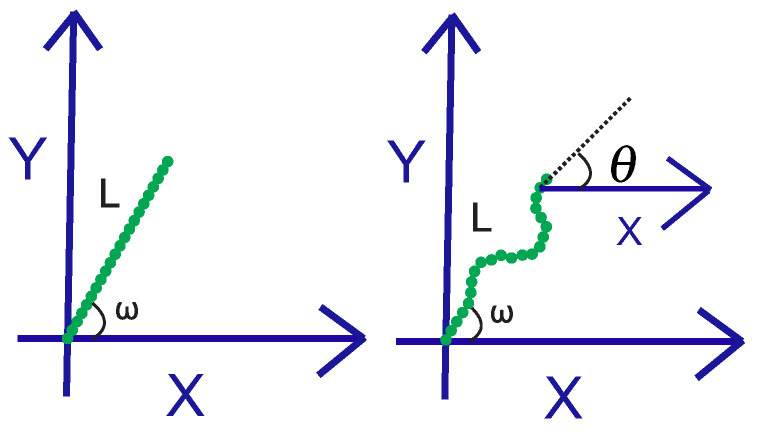
**Left panel:** the zero temperature configuration of the grafted wormlike chain (WLC) in two dimensions. **Right panel:** the grafted filament in the presence of thermal fluctuations. The position and orientation of the free endpoint of the filament fluctuate due to the finite temperature. The filament has a contour length, *L*, and a persistence length, lp, at a finite temperature.

In this section, we review the case of a grafted WLC (in two dimensions, as shown in Figure 1) on the stiff limit. Because of the large value of the bending rigidity, $L\ll l_{P}$, and the deflection away from the grafting direction is small, sin(θ−ω)≈θ−ω and cos(θ−ω)≈1. The positional-orientational propagator of the chain is denoted by $G_{L,l_{p}}\left(x,y,\theta |x_{0},y_{0},\omega \right)$. It is interpreted as the conditional probability density to find the endpoint of the chain at position (x,y) with orientation θ, given that it is grafted at position $\left(x_{0},y_{0}\right)$ with orientation ω.

In the weakly bending regime, the propagator is calculated as a closed analytic expression [47,49,50]

(1) $$\begin{array}{c} G_{L,l_{p}}\left(x,y,\theta |x_{0},y_{0},\omega \right)=\frac{1}{N_{G}}\exp[-\frac{3l_{p}}{L^{3}}(\left(y-y_{0}\right)\cos\left(\omega \right)\\ -\left(x-x_{0}\right)\sin\left(\omega \right){)}^{2}-\frac{l_{p}}{L}{\left(\theta -\omega \right)}^{2}]\\ \times \exp[\frac{3l_{p}}{L^{2}}\left(\left(y-y_{0}\right)\cos\left(\omega \right)-\left(x-x_{0}\right)\sin\left(\omega \right)\right)\left(\theta -\omega \right)]\\ \times \delta [\left(x-x_{0}\right)\cos\left(\omega \right)+\left(y-y_{0}\right)\sin\left(\omega \right)-L]\;, \end{array}$$

where δ(x) is the Dirac δ-function and factor $N_{G}$ is determined by the normalization condition,

(2) $$\int \int \int dxdyd\theta \;G_{L,l_{p}}\left(x,y,\theta |x_{0},y_{0},\omega \right)=1\;.$$

In the remaining of this article, we use the notation $\int \equiv \int_{-\infty }^{+\infty }$ for the sake of simplicity. Using Equation (1), we can easily calculate the probability density of the *x* component of the free endpoint position,

(3) $$\begin{array}{c} P_{x_{1}}\left(x\right)=\int \int dyd\theta G_{L,l_{p}}\left(x,y,\theta |0,0,\omega \right)\\ \;\;\;\;\;\;\;\;\;\;\;=\sqrt{\frac{3l_{p}}{4\pi L^{3}\sin^{2}\left(\omega \right)}}\exp\left(-\frac{3l_{p}{\left(x-L\cos\left(\omega \right)\right)}^{2}}{4L^{3}\sin^{2}\left(\omega \right)}\right)\;. \end{array}$$

The probability density of the *y* component of the free endpoint position is

(4) $$\begin{array}{c} P_{y_{1}}\left(y\right)=\int \int dxd\theta G_{L,l_{p}}\left(x,y,\theta |0,0,\omega \right)\\ \;\;\;\;=\sqrt{\frac{3l_{p}}{4\pi L^{3}\cos^{2}\left(\omega \right)}}\exp\left(-\frac{3l_{p}{\left(y-L\sin\left(\omega \right)\right)}^{2}}{4L^{3}\cos^{2}\left(\omega \right)}\right)\;. \end{array}$$

In addition, the probability density of the orientation of the free endpoint is given by

(5) $$\begin{array}{c} P_{\theta_{1}}\left(\theta \right)=\int \int dxdyG_{L,l_{p}}\left(x,y,\theta |0,0,\omega \right)\\ \;\;\;\;=\sqrt{\frac{l_{p}}{4\pi L}}\exp\left(-\frac{l_{p}{\left(\theta -\omega \right)}^{2}}{4L}\right)\;. \end{array}$$

We point out that even though Equations (3) and (4) rely on the validity of the weakly bending approximation, Equation (5) is exact and holds for any WLC in two dimensions, irrespective of the value of the ratio $l_{l}/L$ [46].

## 3. Grafted Filament with Regular Kinks at the Gaussian Limit

**Figure 2:**
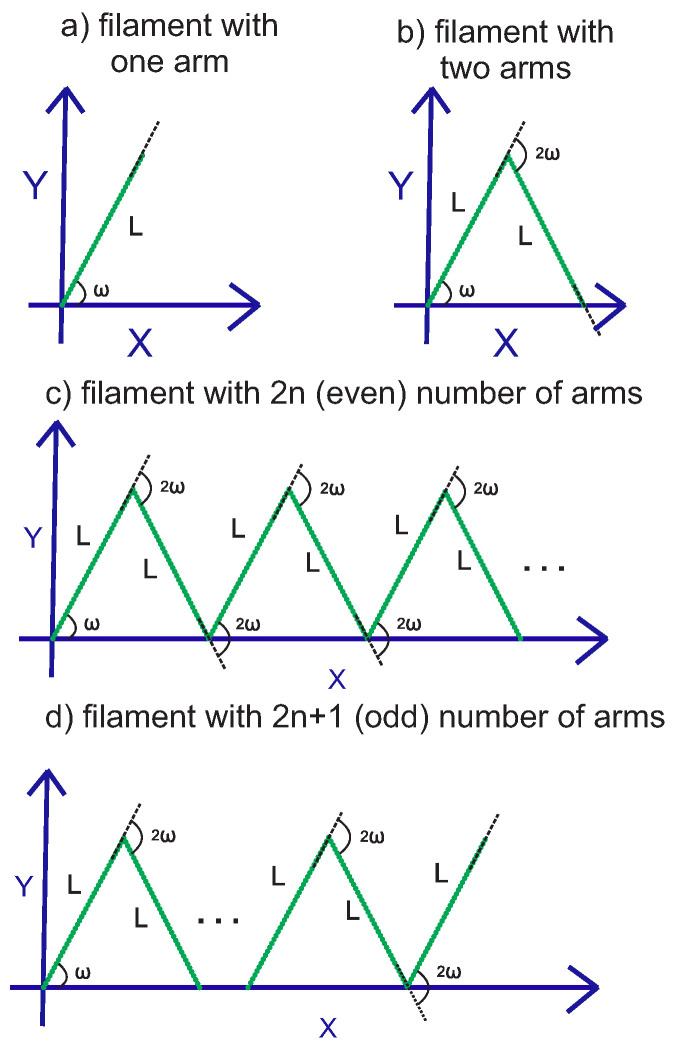
Panel (**a**): the configuration of a grafted filament with one arm (unkinked filament). The grafting angle is ω, and the arm has a contour length *L* and the persistence length $l_{p}$. Panel (**b**): the configuration of the filament with two arms (filament with one kink). Each arm has a contour length *L* and a persistence length $l_{p}$. The grafting angle is ω, and the kink angle is 2ω. Panel (**c**): the configuration of a filament with regular kinks and an even number of similar arms of contour length *L* and persistence length $l_{p}$. Panel (**d**): the configuration of the filament with regular kinks and an odd number of similar arms of the contour lengths *L* and persistence length $l_{p}$. All of the configurations shown in this figure are at zero temperature.

Here, we consider a filament with regular alternating kinks. A kink at one point of the filament is defined as a stiff deviation of the tangent vector at that point by a kink angle. The filament with regular kinks is a zigzag-shaped structure. The structure has a definite number of arms that are linked together at the kinks (see Figure 2). The contour lengths and the persistence lengths of all arms are the same. The number of arms can be even or odd. Due to the specific features of our calculations, we separately study a structure with an even and an odd number of arms.

### 3.1. Filament with an Even Number of Arms

In this subsection, we consider a filament with an even number of arms (see Figure 2). The positional-orientational probability density of the free endpoint of the filament with two arms can be calculated by using twice the positional-orientational propagator in Equation (1),

(6) $$\begin{array}{c} P_{2}\left(x_{2},y_{2},\theta_{2}\right)=\int \int \int G_{L}\left(x_{1},y_{1},\theta_{1}|0,0,\omega \right)\\ \;\;\;\times G_{L}\left(x_{2},y_{2},\theta_{2}|x_{1},y_{1},\theta_{1}-2\omega \right)dx_{1}dy_{1}d\theta_{1} \end{array}$$

Similarly, the positional-orientational probability density of the free endpoint of a filament with four arms is given by applying the propagator four times,

(7) $$\begin{array}{c} P_{4}\left(x_{4},y_{4},\theta_{4}\right)=\int \ldots \int G_{L}\left(x_{1},y_{1},\theta_{1}|0,0,\omega \right)\\ \;\;\;\times G_{L}\left(x_{2},y_{2},\theta_{2}|x_{1},y_{1},\theta_{1}-2\omega \right)\\ \;\;\;\times G_{L}\left(x_{3},y_{3},\theta_{3}|x_{2},y_{2},\theta_{1}+2\omega \right)\\ \;\;\;\times G_{L}\left(x_{4},y_{4},\theta_{4}|x_{3},y_{3},\theta_{3}-2\omega \right)\\ \;\;\;\times dx_{1}dy_{1}d\theta_{1}dx_{2}dy_{2}d\theta_{2}dx_{3}dy_{3}d\theta_{3} \end{array}$$

The positional-orientational probability density of the free endpoint of the filament with 2n arms is obtained by using 2n times the propagator (*n* is an integer number),

(8) $$\begin{array}{c} P_{2n}\left(x_{2n},y_{2n},\theta_{2n}\right)=\int \ldots \int G_{L}\left(x_{1},y_{1},\theta_{1}|0,0,\omega \right)\\ \;\;\;\times G_{L}\left(x_{2},y_{2},\theta_{2}|x_{1},y_{1},\theta_{1}-2\omega \right)\\ \;\;\;\times G_{L}\left(x_{3},y_{3},\theta_{3}|x_{2},y_{2},\theta_{1}+2\omega \right)\\ \;\;\;\times \;\;\;\;\;\;\;\;\;\ldots \\ \;\;\;\times G_{L}\left(x_{2n},y_{2n},\theta_{2n}|x_{2n-1},y_{2n-1},\theta_{2n-1}-2\omega \right)\\ \;\;\;\times dx_{1}dy_{1}d\theta_{1}dx_{2}dy_{2}d\theta_{2}\;\;\ldots \;\;dx_{2n-1}dy_{2n-1}d\theta_{2n-1} \end{array}$$

The positional-orientational probability density of the endpoint of the filament with a number of arms 2n is suggested by a conjecture, and it is proven by mathematical induction in Ref. [31] (see equation number 10 in Ref. [31]),

(9) $$P_{2n}\left(x_{2n},y_{2n},\theta_{2n}\right)\propto \exp\left(A_{2n}{\tilde{\theta }}_{2n}^{2}+B_{2n}{\tilde{\theta }}_{2n}+C_{2n}\right)$$

where

(10) $$A_{2n}=\frac{-7l_{p}}{4(2n)L}$$

and

(11) $$B_{2n}=\frac{3l_{p}}{{\left(2n\right)}^{2}L^{2}}\frac{\left(2n\right)\eta_{x}\cos\left(\omega \right)+\eta_{y}\sin\left(\omega \right)}{\sin(\omega )\cos(\omega )}$$

and

(12) $$C_{2n}=\frac{-3l_{p}}{{\left(2n\right)}^{3}L^{3}}\frac{{\left(2n\right)}^{2}\eta_{x}^{2}\cos{\left(\omega \right)}^{2}+\eta_{y}^{2}\sin{\left(\omega \right)}^{2}}{\sin{\left(\omega \right)}^{2}\cos{\left(\omega \right)}^{2}}$$

Here, we have defined $\eta_{x}=x_{2n}-\left(2n\right)L\cos\left(\omega \right)$, $\eta_{y}=y_{2n}$, and ${\tilde{\theta }}_{2n}=\theta_{2n}+\omega$. We point out that there is a typo in equation number 10 of Ref. [31]. It is corrected here by using ${\tilde{\theta }}_{2n}$ instead of $\theta_{2n}$ in Equation (10) of Ref. [31].

### 3.2. Filament with an Odd Number of Arms

Now, we consider a filament with an odd number of arms (see Figure 2). The positional-orientational probability density of the free endpoint of a filament with 2n+1 arms (*n* is an integer number) is given by applying 2n+1 times the propagator,

(13) $$\begin{array}{l} P_{2n+1}(x_{2n+1},y_{2n+1},\theta_{2n+1})=\\ \;\;\;\int \ldots \int G_{L}\left(x_{1},y_{1},\theta_{1}|0,0,\omega \right)\\ \;\;\;\times G_{L}\left(x_{2},y_{2},\theta_{2}|x_{1},y_{1},\theta_{1}-2\omega \right)\\ \;\;\;\times G_{L}\left(x_{3},y_{3},\theta_{3}|x_{2},y_{2},\theta_{1}+2\omega \right)\\ \;\;\;\times \;\;\;\;\;\;\;\;\;\ldots \\ \;\;\;\times G_{L}\left(x_{2n},y_{2n},\theta_{2n}|x_{2n-1},y_{2n-1},\theta_{2n-1}-2\omega \right)\\ \;\;\;\times G_{L}\left(x_{2n+1},y_{2n+1},\theta_{2n+1}|x_{2n},y_{2n},\theta_{2n}+2\omega \right)\\ \;\;\;\times dx_{1}dy_{1}d\theta_{1}dx_{2}dy_{2}d\theta_{2}\;\;\ldots \;\;dx_{2n}dy_{2n}d\theta_{2n} \end{array}$$

It can be written as one “propagation” of the positional-orientational probability density of the endpoint of the filament with an even (2n) number of arms by the propagator,

(14) $$\begin{array}{l} P_{2n+1}(x_{2n+1},y_{2n+1},\theta_{2n+1})=\\ \;\;\;\int \int \int dx_{2n}dy_{2n}d\theta_{2n}P_{2n}\left(x_{2n},y_{2n},\theta_{2n}\right)\\ \;\;\;\times G_{L}\left(x_{2n+1},y_{2n+1},\theta_{2n+1}|x_{2n},y_{2n},\theta_{2n}+2\omega \right) \end{array}$$

The result of this integration is a closed analytic expression for the positional-orientational probability density of the free endpoint of a filament with 2n+1 arms in the following form:

(15) $$P_{2n+1}\left(x_{2n+1},y_{2n+1},\theta_{2n+1}\right)\propto \exp\left(A_{2n+1}{\tilde{\theta }}_{2n+1}^{2}+B_{2n+1}{\tilde{\theta }}_{2n+1}+C_{2n+1}\right)\;,$$

where

(16) $$A_{2n+1}=\frac{7\left({\left(2n\right)}^{2}+4\;n+\frac{8}{7}\right)l_{p}}{4L\left(1+2n\right)\left({\left(2n\right)}^{2}+4n+2\right)}$$

and

(17) $$B_{2n+1}=\frac{-3\left(\eta_{x}\;\left(1+2n\right)\cos\left(\omega \right)-\sin\left(\omega \right)\eta_{y}\right)l_{p}}{\sin\left(\omega \right)\cos\left(\omega \right)L^{2}\left({\left(2n\right)}^{2}+4n+2\right)}$$

and $C_{2n+1}$ consists of two terms

(18) $$C_{2n+1}=C1_{2n+1}+C2_{2n+1}$$

where the first term is as follows

(19) $$C1_{2n+1}=\frac{-3l_{p}\left(1+2n\right)\left(\eta_{y}+\eta_{x}\;\left(2n\right)+\eta_{x}\right)\left(-\eta_{y}+\eta_{x}\;\left(2n\right)+\eta_{x}\right)}{{\left(\sin\left(\omega \right)\right)}^{2}\left({\left(2n\right)}^{2}+4n+2\right)\left(2n+2\right)\left(2n\right)L^{3}}$$

and the second term is of the following form

(20) $$C2_{2n+1}=\frac{-3l_{p}\left(2\;\sin\left(\omega \right)\cos\left(\omega \right)\eta_{y}\;\eta_{x}+{\eta_{y}}^{2}\left(1+2n\right)\right)}{{\left(\sin\left(\omega \right)\right)}^{2}{\left(\cos\left(\omega \right)\right)}^{2}L^{3}\left(2n\right)\left(2n+2\right)\left({\left(2n\right)}^{2}+4\;n+2\right)}$$

Meanwhile, we have defined $\eta_{x}=x_{2n+1}-\left(2n+1\right)L\cos\left(\omega \right)$, $\eta_{y}=y_{2n+1}-L\sin\left(\omega \right)$, and ${\tilde{\theta }}_{2n+1}=\theta_{2n+1}-\omega$.

For both cases (even and odd numbers of arms), the positional-orientational probability density of the free endpoint is Gaussian with coupled positional-orientational variables. Even though the joint probability density relies on the weakly bending approximation for each arm, the reduced probability density for the orientation of the free endpoint, obtained after integrating the positional variables, is exact, irrespective of the stiffness of the arms (within the WLC model). The reason is that the orientational two-point probability density (orientational propagator) of the WLC is Gaussian, irrespective of the ratio $L/l_{p}$.

## 4. Grafted Weakly Bending Filament with Regular Kinks, with One End Attached to a Magnetic Bead

**Figure 3:**
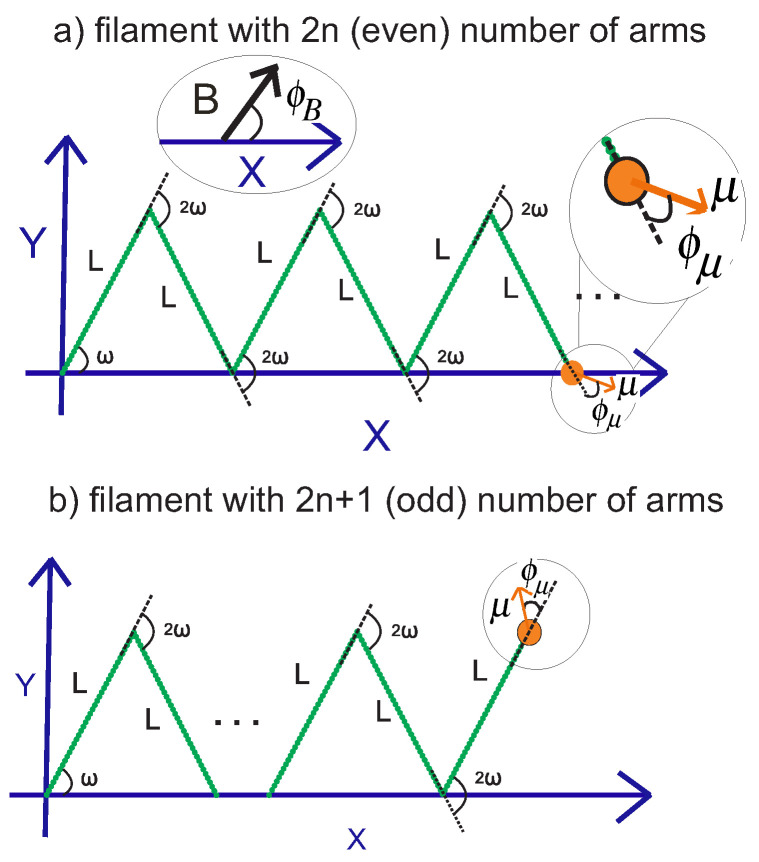
Panel (**a**): The configuration of the grafted kinked filament with an even number of arms with the endpoint attached to a magnetic bead with a magnetic dipole moment in two-dimensional space. Panel (**b**): The configuration of the corresponding system with an odd number of arms. In both panels, there is a constant uniform magnetic field that interacts with the magnetic dipole moment of the magnetic bead. The orientation of the magnetic field relative to the *x*-axis is denoted by $\phi_{B}$. Further, the orientation of the dipole moment of the magnetic bead relative to the orientation of the endpoint of the structure is denoted by $\phi_{\mu }$.

Here, we consider attaching the tip of the grafted semiflexible nanospring to a magnetic bead with a magnetic dipole moment, μ, and expose it to a constant uniform magnetic field, *B*. The energy of the interaction of the magnetic bead with the magnetic field is

(21) $$\kappa_{B}=-\vec{\mu }.\vec{B}=-\mu B\cos\left(\phi \right)$$

where ϕ is the angle between the magnetic dipole of the bead and the magnetic field. The angle, ϕ, is written according to the setup of the model for the structure with an even number of arms (see Figure 3),

(22) $$\phi =\theta_{2n}+\phi_{\mu }-\phi_{B}$$

where $\theta_{2n},\phi_{\mu }$, and $\phi_{B}$ are the orientation of the endpoint of the structure (with 2n arms) with respect to the *x*-axis, the orientation of the magnetic dipole moment of the bead with respect to the orientation of the endpoint of the structure, and the orientation of the magnetic field with respect to the *x*-axis, respectively. Similarly, the angle, ϕ, for the structure with an odd number of arms is of the following form,

(23) $$\phi =\theta_{2n+1}+\phi_{\mu }-\phi_{B}$$

where $\theta_{2n+1}$ is the orientation of the endpoint of the structure (with 2n+1 arms) with respect to the *x*-axis. Meanwhile, we define the ratio of the magnetic energy to the thermal energy, $k_{B}T$, as $K_{B}\cos\left(\phi \right)\equiv \kappa_{B}/\left(k_{B}T\right)$, where $k_{B}$ is the Boltzmann constant.

### 4.1. Filament with an Even Number of Arms

Here, we attach the endpoint of the structure with an even number of arms to a magnetic bead. The positional-orientational probability density of the endpoint of the structure is given by the following closed analytic expression,

(24) $$\begin{array}{l} P_{2n}^{\left(\mu \right)}(x_{2n},y_{2n},\theta_{2n})=\\ \;\;\;P_{2n}\left(x_{2n},y_{2n},\theta_{2n}\right)\\ \;\;\;\times \frac{1}{N_{B}}\exp\left(K_{B}\cos\left(\theta_{2n}+\phi_{\mu }-\phi_{B}\right)\right)\;, \end{array}$$

where $P_{2n}\left(x_{2n},y_{2n},\theta_{2n}\right)$ is given in Equation (9) and $1/N_{B}$ is a normalization prefactor.

The probability density of the orientation of the endpoint of the structure can be calculated by integrating out positional degrees of freedom, $x_{2n}$ and $y_{2n}$,

(25) $$P_{2n}^{\left(\mu \right)}\left(\theta_{2n}\right)=\int \int P_{2n}^{\left(\mu \right)}\left(x_{2n},y_{2n},\theta_{2n}\right)dx_{2n}dy_{2n}=\frac{1}{N_{\theta_{2n}}^{\left(\mu \right)}}\exp\left(A_{\theta_{2n}}^{\mu }\right),$$

where

(26) $$A_{\theta_{2n}}^{\mu }=\left(-\frac{l_{p}{\left(\theta_{2n}+\omega \right)}^{2}}{8nL}+K_{B}\;\cos\left(\theta_{2n}+\varphi_{\mu }-\varphi_{B}\right)\right)$$

and $\frac{1}{N_{\theta_{2n}}^{\left(\mu \right)}}$ is a normalization prefactor. In order to achieve this result, we could have used the orientational propagator for each arm from the beginning (without the positional degrees of freedom). Because the orientational propagator of the WLC does not rely on the weakly bending approximation, this result is exact (within the WLC model), and it holds for filaments of arbitrary stiffness. We point out that because of the cosine in the magnetic interaction, the orientational probability density is not Gaussian. If we assume small fluctuations away from the average value, we recover a Gaussian probability density, but the result shown here is more general.

Now, we implement the weakly bending approximation in Equation (24), where the argument of the exponential function becomes quadratic with respect to all three variables, $x_{2n},y_{2n},\theta_{2n}$. The probability density of the *x* component of the free endpoint of the structure is calculated by integrating the two other variables, namely $\theta_{2n}$ and $y_{2n}$,

(27) $$P_{2n}^{\left(\mu \right)}\left(x_{2n}\right)=\int \int P_{2n}^{\left(\mu \right)}\left(x_{2n},y_{2n},\theta_{2n}\right)dy_{2n}d\theta_{2n}$$

The result of the integration is a Gaussian probability density of the *x* component of the endpoint position of the structure with an even number of arms,

(28) $$P_{2n}^{\left(\mu \right)}\left(x_{2n}\right)=\frac{1}{N_{x_{2n}}^{\left(\mu \right)}}\exp\left(\alpha_{x_{2n}}^{\left(\mu \right)}{\left(x_{2n}+\beta_{x_{2n}}^{\left(\mu \right)}\right)}^{2}\right)\;,$$

where

(29) $$\alpha_{x_{2n}}^{\left(\mu \right)}=\frac{-3l_{p}\left(K_{B}\;\cos\left(\omega -\varphi_{\mu }+\varphi_{B}\right)\left(2n\right)L+1/2\;l_{p}\right)}{{\left(\sin\left(\omega \right)\right)}^{2}\left(2\;l_{p}+K_{B}\;\cos\left(\omega -\varphi_{\mu }+\varphi_{B}\right)\left(2n\right)L\right)\left(2n\right)L^{3}}$$

and

(30) $$\begin{array}{l} \beta_{x_{2n}}^{\left(\mu \right)}=-\frac{L^{2}{\left(2n\right)}^{2}K_{B}\;\cos\left(\omega -\varphi_{\mu }+\varphi_{B}\right)\cos\left(\omega \right)}{K_{B}\;\cos\left(\omega -\varphi_{\mu }+\varphi_{B}\right)\left(2n\right)L+1/2\;l_{p}}\\ \;\;\;-\;\frac{L\left(LK_{B}\;\sin\left(\omega \right)\sin\left(\omega -\varphi_{\mu }+\varphi_{B}\right)+l_{p}\cos\left(\omega \right)\right)\left(2n\right)}{2K_{B}\;\cos\left(\omega -\varphi_{\mu }+\varphi_{B}\right)\left(2n\right)L+l_{p}} \end{array}$$

Similarly, we calculate the probability density of the *y* component of the free endpoint position of the structure with an even number of arms,

(31) $$P_{2n}^{\left(\mu \right)}\left(y_{2n}\right)=\int \int P_{2n}^{\left(\mu \right)}\left(x_{2n},y_{2n},\theta_{2n}\right)dx_{2n}d\theta_{2n}$$

The resulting probability density is the following Gaussian expression,

(32) $$P_{2n}^{\left(\mu \right)}\left(y_{2n}\right)=\frac{1}{N_{y_{2n}}^{\left(\mu \right)}}\exp\left(\alpha_{y_{2n}}^{\left(\mu \right)}{\left(y_{2n}+\beta_{y_{2n}}^{\left(\mu \right)}\right)}^{2}\right)\;,$$

where

(33) $$\alpha_{y_{2n}}^{\left(\mu \right)}=\frac{-3l_{p}\left(K_{B}\;\cos\left(\omega -\varphi_{\mu }+\varphi_{B}\right)\left(2n\right)L+1/2\;l_{p}\right)}{\left(2\;l_{p}+K_{B}\;\cos\left(\omega -\varphi_{\mu }+\varphi_{B}\right)\left(2n\right)L\right){\left(\cos\left(\omega \right)\right)}^{2}{\left(2n\right)}^{3}L^{3}}\;,$$

and

(34) $$\beta_{y_{2n}}^{\left(\mu \right)}=\;\frac{-K_{B}\;{\left(2n\right)}^{2}L^{2}\sin\left(\omega -\varphi_{\mu }+\varphi_{B}\right)\cos\left(\omega \right)}{2K_{B}\;\cos\left(\omega -\varphi_{\mu }+\varphi_{B}\right)\left(2n\right)L+l_{p}}$$

### 4.2. Filament with an Odd Number of Arms

Now, we consider the structure with an odd number of arms and a magnetic bead to the free endpoint of the grafted structure. As in the case with an even number of arms, the joint positional-orientational probability density for the free end has the form

(35) $$\begin{array}{l} P_{2n+1}^{\left(\mu \right)}(x_{2n+1},y_{2n+1},\theta_{2n+1})=\\ \;\;\;P_{2n+1}\left(x_{2n+1},y_{2n+1},\theta_{2n+1}\right)\\ \;\;\;\times \frac{1}{N_{B}^{\prime }}\exp\left(K_{B}\cos\left(\theta_{2n+1}+\phi_{\mu }-\phi_{B}\right)\right), \end{array}$$

where $P_{2n+1}\left(x_{2n+1},y_{2n+1},\theta_{2n+1}\right)$ is given in Equation (15) and $1/N_{B}^{\prime }$ is a normalization prefactor. As before, we obtain the three reduced probability densities.

The probability density of the orientation of the endpoint of the structure is

(36) $$P_{2n+1}^{\left(\mu \right)}\left(\theta_{2n+1}\right)=\frac{1}{N_{\theta_{2n+1}}^{\left(\mu \right)}}\exp\left(A_{\theta_{2n+1}}^{\mu }\right)$$

where

(37) $$A_{\theta_{2n+1}}^{\mu }=\left(-\frac{l_{p}{\left(\theta_{2n+1}-\omega \right)}^{2}}{4(2n+1)L}+K_{B}\;\cos\left(\theta_{2n+1}+\varphi_{\mu }-\varphi_{B}\right)\right)$$

This result, which could have also been obtained by using the orientational propagator from the beginning, is exact (within the WLC model) and holds for a nanospring of arbitrary stiffness.

As before, we implement the Gaussian (weakly bending approximation) for all three variables and integrate the angle in order to get the reduced positional probabilities. The probability density of the *x* component of the endpoint position is

(38) $$P_{2n+1}^{\left(\mu \right)}\left(x_{2n+1}\right)=\frac{1}{N_{x_{2n+1}}^{\left(\mu \right)}}\exp\left(\alpha_{x_{2n+1}}^{\left(\mu \right)}{\left(x_{2n+1}+\beta_{x_{2n+1}}^{\left(\mu \right)}\right)}^{2}\right)$$

where

(39) $$\alpha_{x_{2n+1}}^{\left(\mu \right)}=\frac{-3\;\left(LK_{B}\;\left(2n+1\right)\cos\left(\omega +\varphi_{\mu }-\varphi_{B}\right)+1/2\;l_{p}\right)l_{p}}{\alpha_{1}}$$

and

(40) $$\begin{array}{r} \alpha_{1}=+{\left(\sin\left(\omega \right)\right)}^{2}{\left(2n+1\right)}^{2}L^{4}K_{B}\;\cos\left(\omega +\varphi_{\mu }-\varphi_{B}\right)\\ +2\;{\left(\sin\left(\omega \right)\right)}^{2}\left(2n+1\right)l_{p}L^{3} \end{array}$$

and

(41) $$\begin{array}{l} \beta_{x_{2n+1}}^{\left(\mu \right)}=-2\;\frac{L^{2}\cos\left(\omega \right)K_{B}\;{\left(2n+1\right)}^{2}\cos\left(\omega +\varphi_{\mu }-\varphi_{B}\right)}{2\;LK_{B}\;\left(2n+1\right)\cos\left(\omega +\varphi_{\mu }-\varphi_{B}\right)+l_{p}}\\ \;\;\;\;\;-\frac{l_{p}\cos\left(\omega \right)\left(2n+1\right)L}{2\;LK_{B}\;\left(2n+1\right)\cos\left(\omega +\varphi_{\mu }-\varphi_{B}\right)+l_{p}}\\ \;\;\;\;\;-\frac{L^{2}\sin\left(\omega \right)K_{B}\;\sin\left(\omega +\varphi_{\mu }-\varphi_{B}\right)\left(2n+1\right)}{2\;LK_{B}\;\left(2n+1\right)\cos\left(\omega +\varphi_{\mu }-\varphi_{B}\right)+l_{p}} \end{array}$$

Similarly, the probability density of the *y* component of the free endpoint position is

(42) $$P_{2n+1}^{\left(\mu \right)}\left(y_{2n+1}\right)=\frac{1}{N_{y_{2n+1}}^{\left(\mu \right)}}\exp\left(\alpha_{y_{2n+1}}^{\left(\mu \right)}{\left(y_{2n+1}+\beta_{y_{2n+1}}^{\left(\mu \right)}\right)}^{2}\right)$$

where

(43) $$\alpha_{y_{2n+1}}^{\left(\mu \right)}=\frac{-3\;\left(LK_{B}\;\left(2n+1\right)\cos\left(\omega +\varphi_{\mu }-\varphi_{B}\right)+1/2\;l_{p}\right)l_{p}}{\alpha_{2}}$$

and

(44) $$\begin{array}{r} \alpha_{2}={\left(\cos\left(\omega \right)\right)}^{2}{\left(2n+1\right)}^{4}L^{4}K_{B}\;\cos\left(\omega +\varphi_{\mu }-\varphi_{B}\right)\\ +2\;{\left(\cos\left(\omega \right)\right)}^{2}{\left(2n+1\right)}^{3}L^{3}l_{p} \end{array}$$

and

(45) $$\begin{array}{r} \beta_{y_{2n+1}}^{\left(\mu \right)}=\frac{L^{2}K_{B}\;\cos\left(\omega \right){\left(2n+1\right)}^{2}\sin\left(\omega +\varphi_{\mu }-\varphi_{B}\right)}{2LK_{B}\;\left(2n+1\right)\cos\left(\omega +\varphi_{\mu }-\varphi_{B}\right)+l_{p}}\\ -L\sin\left(\omega \right) \end{array}$$

**Figure 4:**
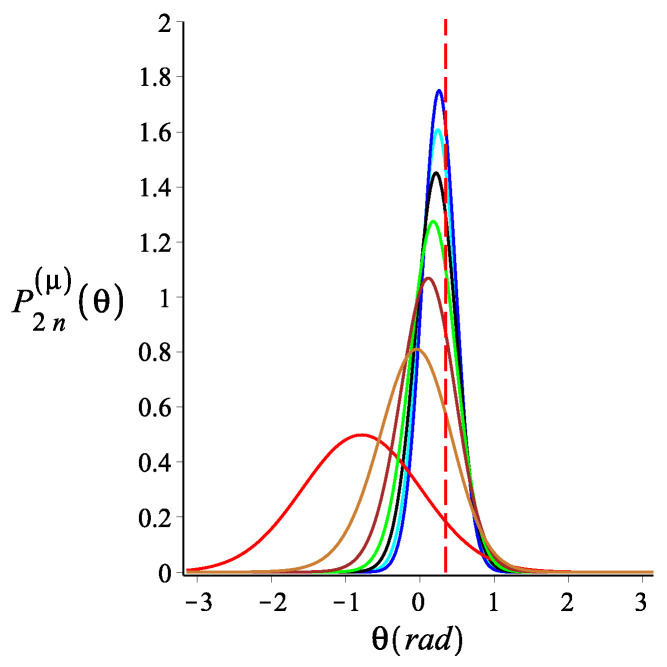
The probability density of the orientation of the free endpoint of the grafted structure with an even number of arms. The red, gold, brown, green, black, cyan, and blue are associated with the fixed value of the magnetic energy parameter, $K_{B}=0,3,6,9,12,15,18$, respectively. The other fixed parameters are: $\omega =\frac{\pi }{4}$, $\phi_{\mu }=0$, $\phi_{B}=\frac{\pi }{9}$n=2, and $\frac{l_{p}}{L}=12.5$. The red dashed line is associated with $\theta =\frac{\pi }{9}$, which is also the value of $\phi_{B}$.

**Figure 5:**
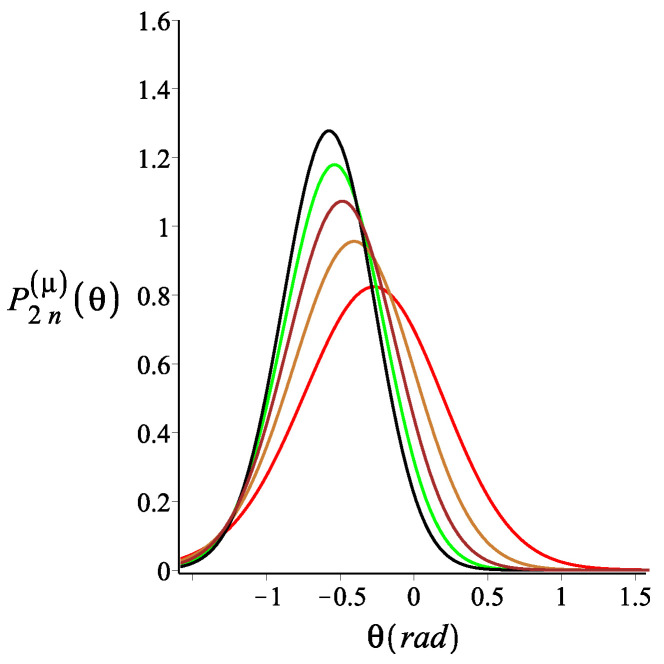
The probability density of the orientation of the free endpoint of the grafted structure with an even number of arms. The red, gold, brown, green, and black are associated with the fixed values for the stiffness parameter, $\frac{l_{p}}{L}=12.5i$, where i=1,2,3,4,5, respectively. The other fixed parameters are: $\omega =\frac{\pi }{4}$, $\phi_{\mu }=0$, $\phi_{B}=0$n=2, $K_{B}=3$.

**Figure 6:**
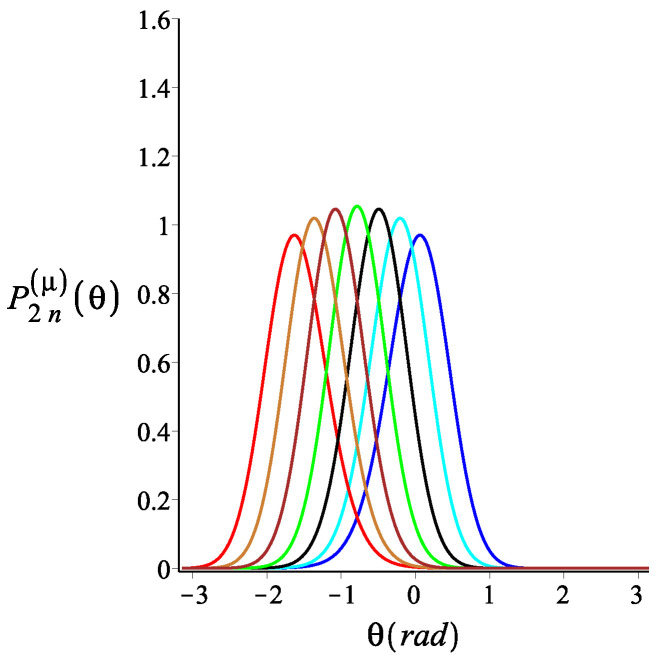
The probability density of orientation of the free endpoint of the grafted structure with an even number of arms. The red, gold, brown, green, black, cyan, and blue are associated with the fixed values for the orientation of the external magnetic field, $\phi_{B}=-\omega -\frac{\pi }{2}+\frac{\pi }{6}i$, where i=0,1,2,3,4,5,6, respectively. The other fixed parameters are: $\omega =\frac{\pi }{4}$, $\phi_{\mu }=0$, $\frac{l_{p}}{L}=12.5$, n=1, and $K_{B}=4$.

In Figure 4, Figure 5 and Figure 6, we show how the orientation of the free end changes as we change the strength of the magnetic interaction, the bending stiffness of the arms, and the orientation of the external field, respectively. We notice that by increasing the strength of the magnetic interaction, not only does the average orientation of the free end change, but the fluctuations (the width of the distribution) also decrease. Keeping all the other parameters fixed, changing the bending stiffness changes both the average orientation of the free end and the width of its fluctuations. Similarly, rotating the external field changes both the average direction of the free end and its fluctuations.

**Figure 7:**
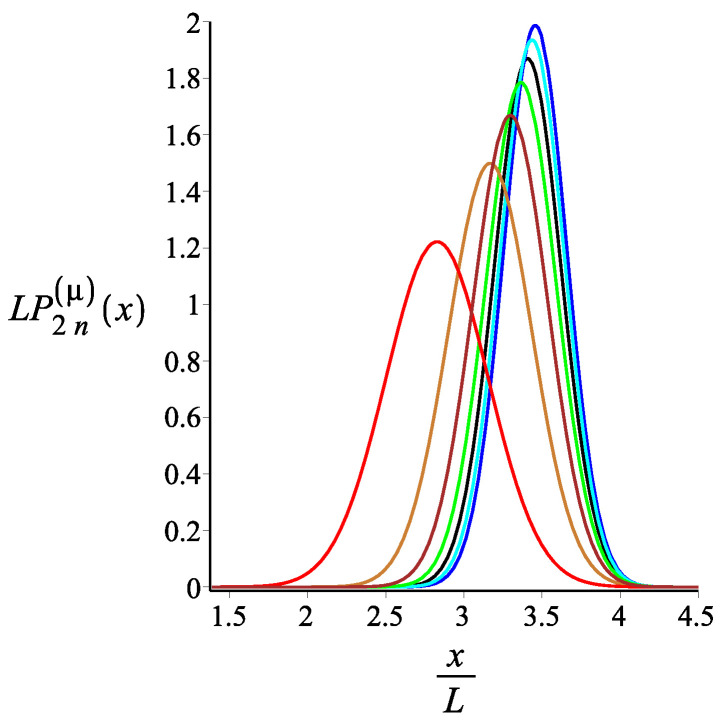
The probability density of the *x* component of the position of the free endpoint of the grafted structure with an even number of arms. The red, gold, brown, green, black, cyan, and blue are associated with the fixed values of the magnetic interaction parameter, $K_{B}=0,3,6,9,12,15,18$, respectively. The other fixed parameters are: $\omega =\frac{\pi }{4}$, $\phi_{\mu }=0$, $\phi_{B}=\frac{\pi }{9}$n=2, and $\frac{l_{p}}{L}=12.5$.

**Figure 8:**
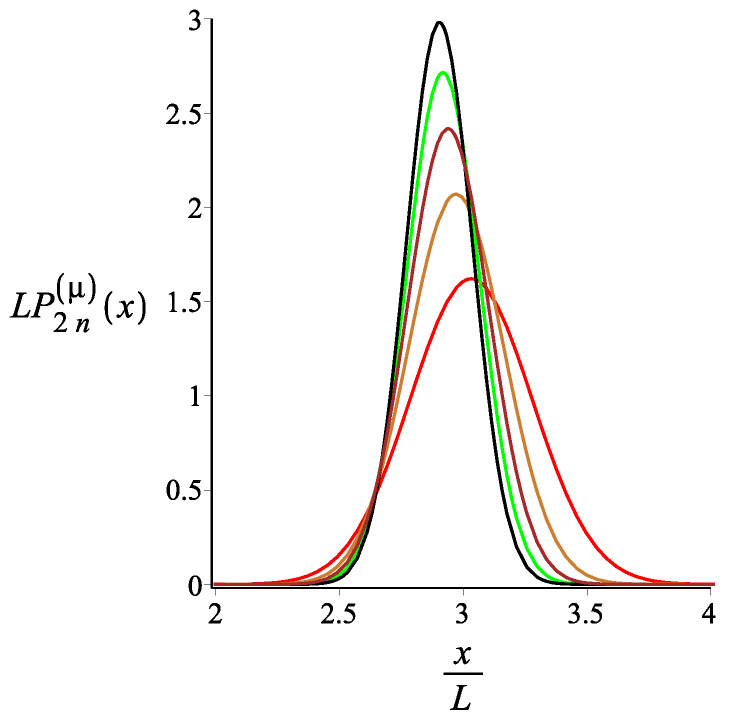
The probability density of the *x* component of the position of the free endpoint of the grafted structure with an even number of arms. The red, gold, brown, green, and black are associated with the fixed values of the stiffness parameter, $\frac{l_{p}}{L}=12.5i$, where i=1,2,3,4,5, respectively. The other fixed parameters are: $\omega =\frac{\pi }{4}$, $\phi_{\mu }=0$, $\phi_{B}=0$n=2, and $K_{B}=3$.

**Figure 9:**
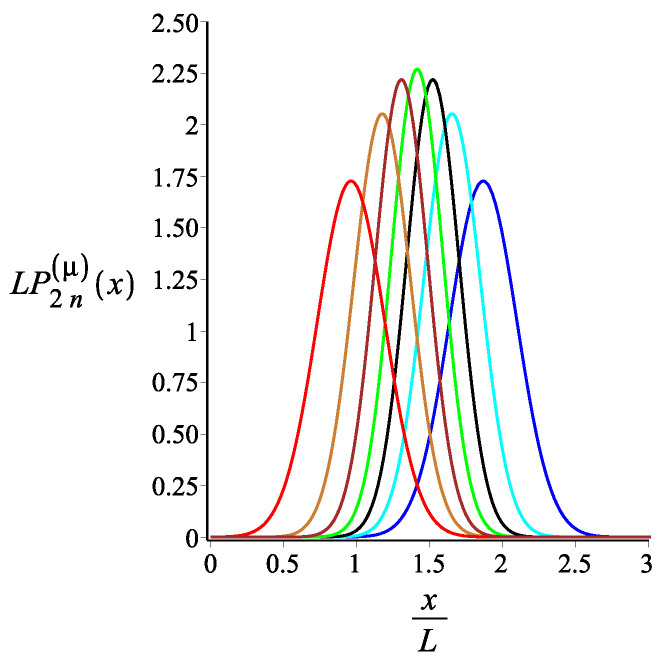
The probability density of the *x* component of the position of the free endpoint of the structure with an even number of arms. The red, gold, brown, green, black, cyan, and blue are associated with the fixed values of the orientation of the external magnetic field, $\phi_{B}=-\omega -\frac{\pi }{2}+\frac{\pi }{6}i$, where i=0,1,2,3,4,5,6, respectively. The other fixed parameters are: $\omega =\frac{\pi }{4}$, $\phi_{\mu }=0$, $\frac{l_{p}}{L}=12.5$, n=1, and $K_{B}=4$.

**Figure 10:**
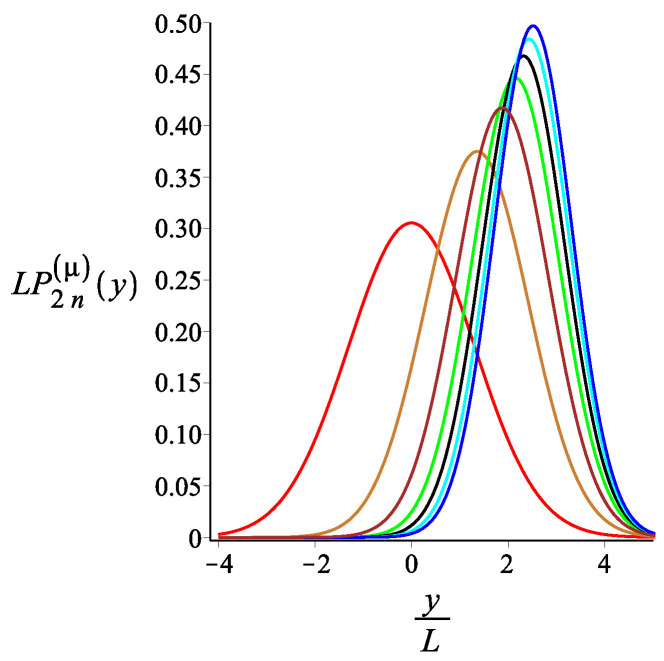
The probability density of the *y* component of the position of the free endpoint of the grafted structure with an even number of arms. The red, gold, brown, green, black, cyan, and blue are associated with the fixed values of the magnetic interaction parameter, $K_{B}=0,3,6,9,12,15,18$, respectively. The other fixed parameters are: $\omega =\frac{\pi }{4}$, $\phi_{\mu }=0$, $\phi_{B}=\frac{\pi }{9}$n=2, and $\frac{l_{p}}{L}=12.5$.

**Figure 11:**
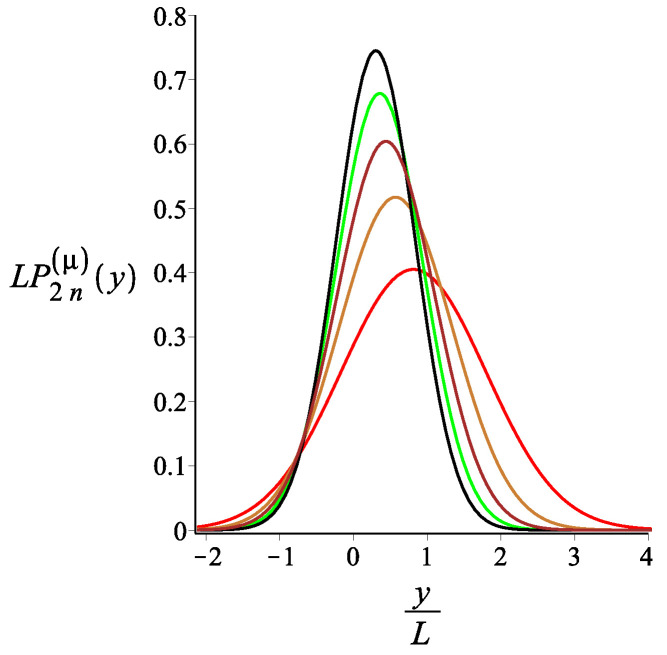
The probability density of the *y* component of the position of the free endpoint of the grafted structure with an even number of arms. The red, gold, brown, green, and black are associated with the fixed values of the stiffness parameter, $\frac{l_{p}}{L}=12.5i$, where i=1,2,3,4,5, respectively. The other fixed parameters are: $\omega =\frac{\pi }{4}$, $\phi_{\mu }=0$, $\phi_{B}=0$n=2, and $K_{B}=3$.

**Figure 12:**
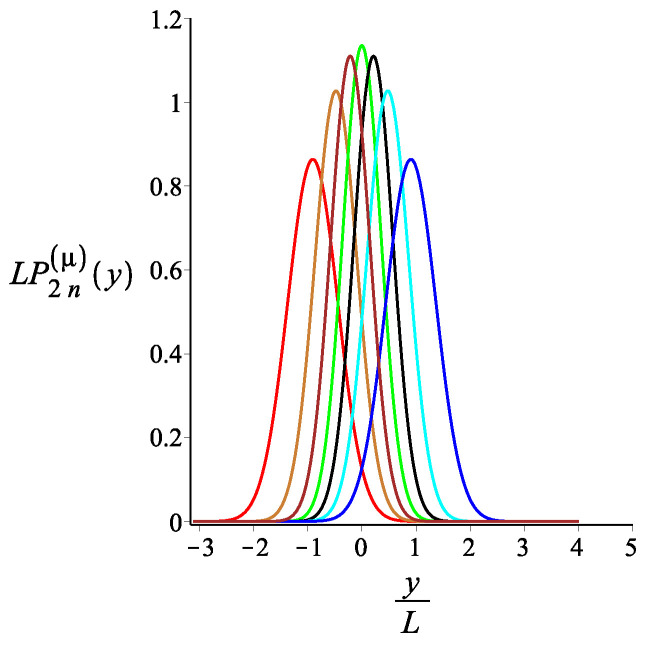
The probability density of the *y* component of the position of the free endpoint of the structure with an even number of arms. The red, gold, brown, green, black, cyan, and blue are associated with the fixed values of the orientation of the external magnetic field, $\phi_{B}=-\omega -\frac{\pi }{2}+\frac{\pi }{6}i$, where i=0,1,2,3,4,5,6, respectively. The other fixed parameters are: $\omega =\frac{\pi }{4}$, $\phi_{\mu }=0$, $\frac{l_{p}}{L}=12.5$, n=1, and $K_{B}=4$.

In Figure 7, Figure 8 and Figure 9, we show how the *x* component of the position of the free end changes as we change the strength of the magnetic interaction, the bending stiffness of the arms, and the orientation of the external field, respectively. Remarkably, because of the zigzag geometry of the structure, just by changing the strength or the orientation of the external magnetic field, we can change the extension of the spring (the distance of the free end from the grafting substrate). The corresponding behavior of the *y* component is shown in Figure 10, Figure 11 and Figure 12. We point out that in both directions, *x* and *y*, we receive similar behavior, and the spring is more or less equally responsive.

## 5. Force-Extension Relation in the Longitudinal and Transverse Direction

The Helmholtz free energy associated with the probability density of the free endpoint position of the filament in the *x* coordinate, $P_{x}\left(x\right)$, is

(46) $$F_{x}\left(x\right)=-k_{B}T\ln\left(P_{x}\left(x\right)\right)$$

The resulting force-extension relation associated with the endpoint of the filament in the *x* coordinate in the fixed-extension (isometric or Helmholtz) ensemble is obtained by taking the derivative of the free energy with respect to *x*,

(47) $$\langle f_{x}\rangle =\frac{dF_{x}\left(x\right)}{dx}=-k_{B}T\frac{d}{dx}\ln\left(P_{x}\left(x\right)\right)$$

The stiffness (elastic constant) in the *x*-direction is defined as

(48) $$E_{x}=\frac{d\langle f_{x}\rangle }{dx}$$

Similarly, the force-extension relation associated with the *y* coordinate of the filament tip in the fixed-extension (isometric or Helmholtz) ensemble is obtained as follows,

(49) $$\langle f_{y}\rangle =\frac{dF_{y}\left(y\right)}{dy}=-k_{B}T\frac{d}{dy}\ln\left(P_{y}\left(y\right)\right)$$

Further, the stiffness in the *y*-direction is as follows,

(50) $$E_{y}=\frac{d\langle f_{y}\rangle }{dy}$$

In order to be consistent with the notation that we introduced in the previous sections, we separately calculate the force-extension relations and the related elastic constants for springs with an even and odd number of arms.

### 5.1. Filament with an Even Number of Arms

The force-extension relation in the *x*-direction of the tip of a grafted kinked filament with an even (2n) number of arms is

(51) $$\langle f_{x_{2n}}\rangle =-2k_{B}T\alpha_{x_{2n}}^{\left(\mu \right)}\left(x_{2n}+\beta_{x_{2n}}^{\left(\mu \right)}\right)$$

The elastic constant in the *x*-direction is

(52) $$E_{x_{2n}}=-2k_{B}T\alpha_{x_{2n}}^{\left(\mu \right)}$$

Similarly, the force-extension relation in the *y*-direction for the tip of a grafted kinked filament with 2n arms is

(53) $$\langle f_{y_{2n}}\rangle =-2k_{B}T\alpha_{y_{2n}}^{\left(\mu \right)}\left(y_{2n}+\beta_{y_{2n}}^{\left(\mu \right)}\right)$$

The force constant in the *y*-direction is

(54) $$E_{y_{2n}}=-2k_{B}T\alpha_{y_{2n}}^{\left(\mu \right)}$$

### 5.2. Filament with an Odd Number of Arms

The force-extension relation in the *x*-direction for a kinked filament with an odd (2n+1) number of arms is

(55) $$\langle f_{x_{2n+1}}\rangle =-2k_{B}T\alpha_{x_{2n+1}}^{\left(\mu \right)}\left(x_{2n+1}+\beta_{x_{2n+1}}^{\left(\mu \right)}\right)$$

The force constant in the *x*-direction is

(56) $$E_{x_{2n+1}}=-2k_{B}T\alpha_{x_{2n+1}}^{\left(\mu \right)}$$

The force-extension relation in the *y*-direction is

(57) $$\langle f_{y_{2n+1}}\rangle =-2k_{B}T\alpha_{y_{2n+1}}^{\left(\mu \right)}\left(y_{2n+1}+\beta_{y_{2n+1}}^{\left(\mu \right)}\right)$$

The corresponding elastic constant in the *y*-direction is

(58) $$E_{y_{2n+1}}=-2k_{B}T\alpha_{y_{2n+1}}^{\left(\mu \right)}$$

In the weakly bending approximation, these force-extension relations are linear. The interesting point is that the elastic constants depend on many parameters, and this allows us to control the elasticity of the spring in many different ways. They depend on the stiffness of the arms ($l_{p}/L$), the kink angle (2ω), the number of arms (2n or 2n+1), the strength of the magnetic interaction ($K_{B}$), and the orientation of the external field ($\phi_{B}$).

**Figure 13:**
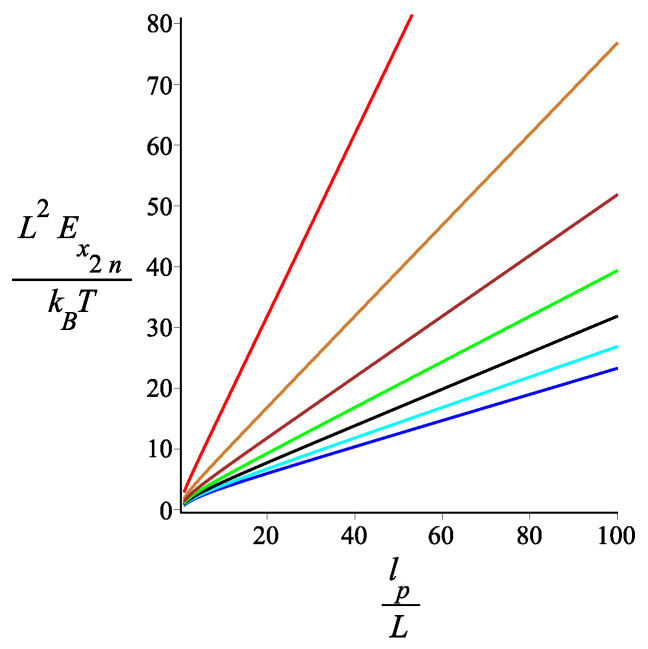
The dimensionless tensile stiffness of the grafted kinked structure with an even number of arms as a function of the dimensionless persistence length of a single arm. The red, gold, brown, green, black, cyan, and blue correspond to fixed values of the parameter n=1,2,3,4,5,6,7, respectively. The other fixed parameters are: $\omega =\frac{\pi }{4}$, $\phi_{\mu }=0$, $\phi_{B}=\frac{\pi }{9}$n=2, and $K_{B}=1$.

In Figure 13, we see the dependence of the tensile stiffness of the spring on the bending stiffness of the arms and on the number of arms. As expected, the filament becomes softer as we increase the number of arms. For large *n*, the stiffness decreases as 1/n.

**Figure 14:**
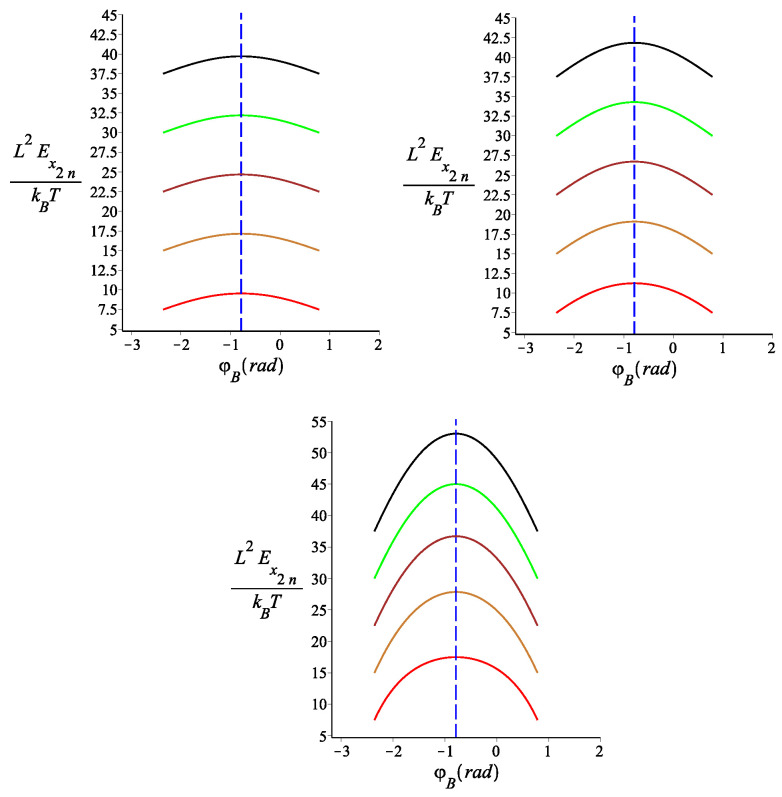
The dimensionless tensile stiffness of the structure with an even number of arms as a function of the orientation of the external magnetic field. The red, gold, brown, green, and black are associated with fixed values of the single-arm bending stiffness parameter $\frac{l_{p}}{L}=10,20,30,40,50$, respectively. The other fixed parameters are: $\omega =\frac{\pi }{4}$, $\phi_{\mu }=0$, and n=2. For the top left panel, we have $K_{B}=0.5$. For the top right panel, we have $K_{B}=1$. For the lower panel, we have $K_{B}=4$.

One of the most interesting results of our analysis is the sensitivity of the tensile stiffness on the orientation of the external field, as shown in Figure 14.

In this section, we calculate the force-extension relations in the Helmholtz (fixed-extension) ensemble. However, the same results hold in the Gibbs (isotensional, fixed force) ensemble. As long as the fluctuations of the tip position away from the average are Gaussian (weakly bending approximation), the response is linear, and this implies ensemble equivalence [51].

## 6. Force Exerted on a Stiff Planar Wall by the Tip of the Kinked Filament

**Figure 15:**
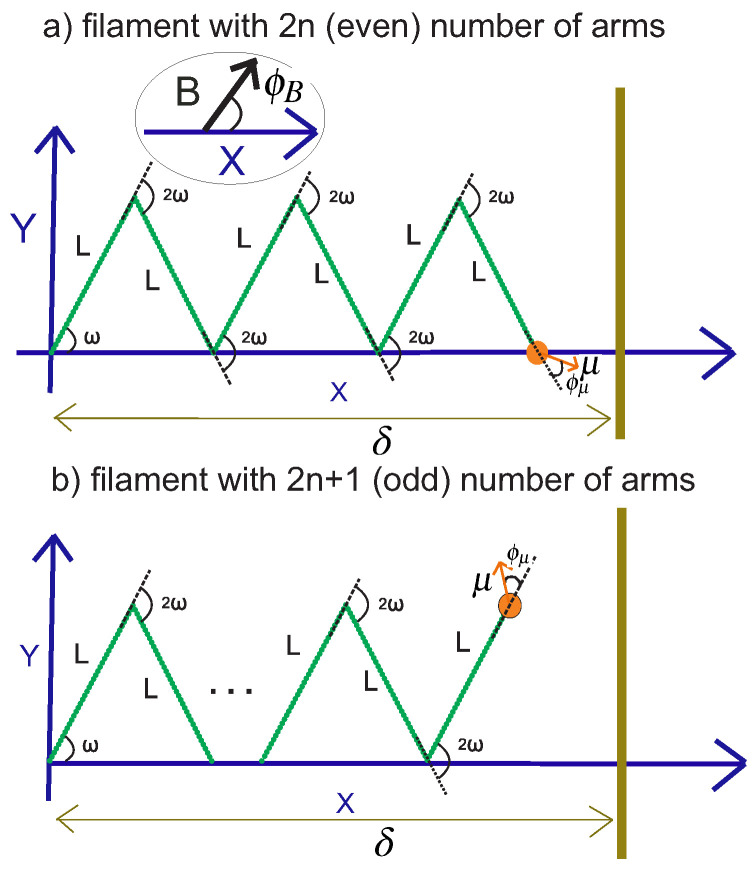
Panel (**a**): The endpoint of the kinked filament with an even number of arms is attached to the magnetic bead and is confined by a rigid impenetrable wall in the *x*-direction. Panel (**b**): The similar confined system with an odd number of arms.

In this section, we consider the grafted kinked structure with the magnetic moment at the tip, and that is confined by a stiff and impenetrable wall in the *x*-direction. The distance of the wall from the grafting point of the structure is fixed, δ, while the fluctuating endpoint of the kinked structure exerts a fluctuating force on the wall (see Figure 15).

The force-extension relation of this system refers to the average force exerted by the fluctuating tip of the filament on the wall as a function of the confining distance δ. In order to calculate it, we use the method introduced in Refs. [47,48,52]. We view this force as entropic, originating in the reduction of the number of configurations of the system due to the presence of the confining wall. The number of configurations of the system is proportional to the probability of the *x* coordinate of the tip being within the confining region 0<x<δ,

(59) $$Z\left(\delta \right)=\int_{0}^{\delta }P_{x}\left(x\right)dx$$

where $P_{x}\left(x\right)$ is the probability density to find the *x* component of the endpoint position of the kinked filament at the value *x*. We obtain the average force of the polymer tip on the wall by calculating the derivative of the logarithm of the number of the configuration of the system with respect to δ,

(60) $$\langle f_{x}^{PW}\left(\delta \right)\rangle =k_{B}T\frac{d}{d\delta }\ln\left(Z\left(\delta \right)\right)$$

We note that this expression differs from the corresponding one for the tensile force that we calculated in the previous section by a minus sign. The reason is that when we increase the *x* (or the *y* component) of the free tip, the entropy of the grafted zigzag spring decreases, but as we increase the confining distance δ, the entropy increases. It is obvious that we ignore the steric effect of the wall on the rest of the system (apart from the tip) in this method. However, the error is expected to be small for small compression. The differential stiffness of the compressed structure can be calculated from the slope of the force-extension relation,

(61) $$E_{x}^{PW}=-\frac{d\langle f_{x}^{PW}\left(\delta \right)\rangle }{d\delta }$$

In the case of a kinked filament with an even number of arms, 2n, we insert into Equation (59) $P_{x_{2n}}^{\left(\mu \right)}\left(x\right)$ for the probability density of the *x* component of free endpoint position. The resulting average force is

(62) $$\langle f_{x_{2n}}^{PW}\left(\delta \right)\rangle =-\frac{2k_{B}T}{A_{2n}^{PW}}\frac{\alpha_{x_{2n}}^{\left(\mu \right)}}{\sqrt{-\pi \alpha_{x_{2n}}^{\left(\mu \right)}}}\exp\left(\frac{{\left(2\;\alpha_{x_{2n}}^{\left(\mu \right)}\;\delta +\beta_{x_{2n}}^{\left(\mu \right)}\right)}^{2}}{4\alpha_{x_{2n}}^{\left(\mu \right)}}\right)$$

where

(63) $$A_{2n}^{PW}=\left(\mathrm{erf}\left(\frac{\beta_{x_{2n}}^{\left(\mu \right)}}{2\sqrt{-\alpha_{x_{2n}}^{\left(\mu \right)}}}\right)-\mathrm{erf}\left(\frac{2\;\alpha_{x_{2n}}^{\left(\mu \right)}\;\delta +\beta_{x_{2n}}^{\left(\mu \right)}}{2\sqrt{-\alpha_{x_{2n}}^{\left(\mu \right)}}}\right)\right)$$

The differential stiffness of this system is

(64) $$E_{x_{2n}}^{PW}=-\frac{d\langle f_{x_{2n}}^{PW}\left(\delta \right)\rangle }{d\delta }$$

We can easily obtain the corresponding expressions for a system with an odd number of arms by simply replacing 2n in the previous expressions with 2n+1. (erf(a) is the error function of *a*.) We obtain an analytic expression for the force-extension relation because the calculation is based on the Gaussian form for the positional probability density of the free tip, which, in turn, relies on the weakly bending approximation.

**Figure 16:**
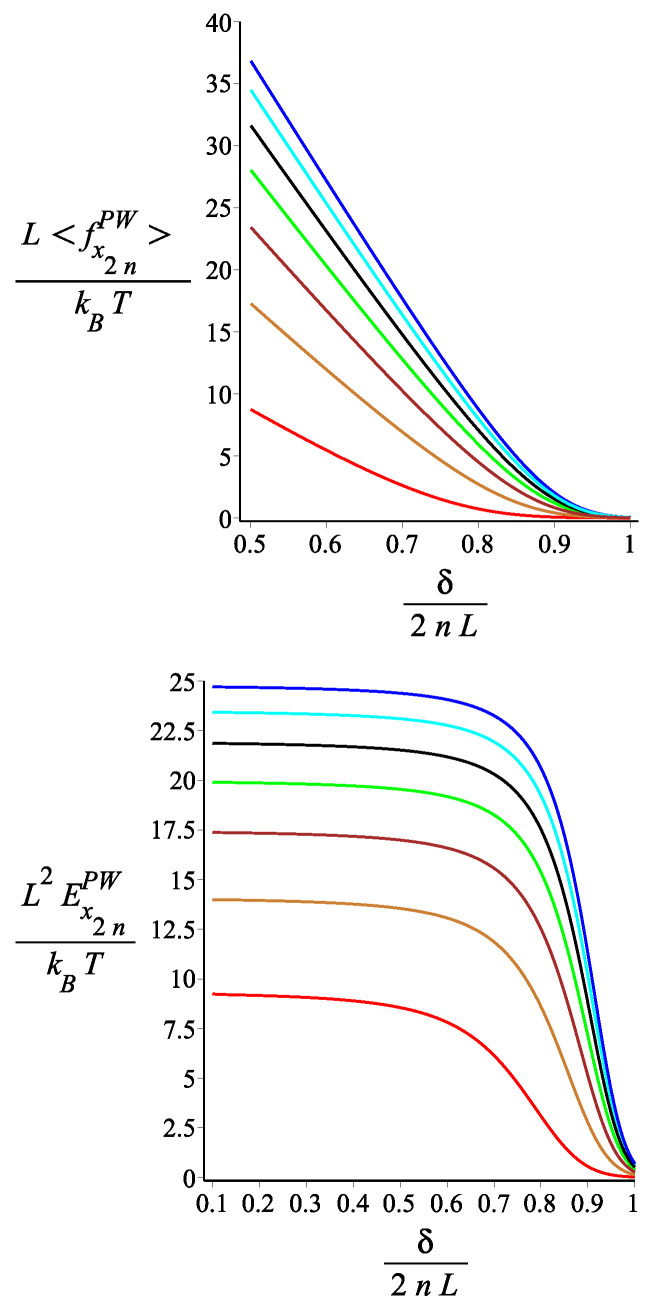
The **first panel**: The dimensionless force of the structure with an even number of arms exerted on the confining wall as a function of the dimensionless distance of the wall from the grafting point. The **second panel**: The dimensionless differential stiffness of the system with an even number of arms as a function of the dimensionless distance of the wall from the grafting point. The red, gold, brown, green, black, cyan, and blue are associated with the fixed values for the magnetic interaction $K_{B}=0,3,6,9,12,15,18$, respectively. The other fixed parameters are: $\omega =\frac{\pi }{4}$, $\phi_{\mu }=0$, $\phi_{B}=\frac{\pi }{9}$n=2, and $\frac{l_{p}}{L}=12.5$. The red dashed line is associated with $\theta =\frac{\pi }{9}$.

**Figure 17:**
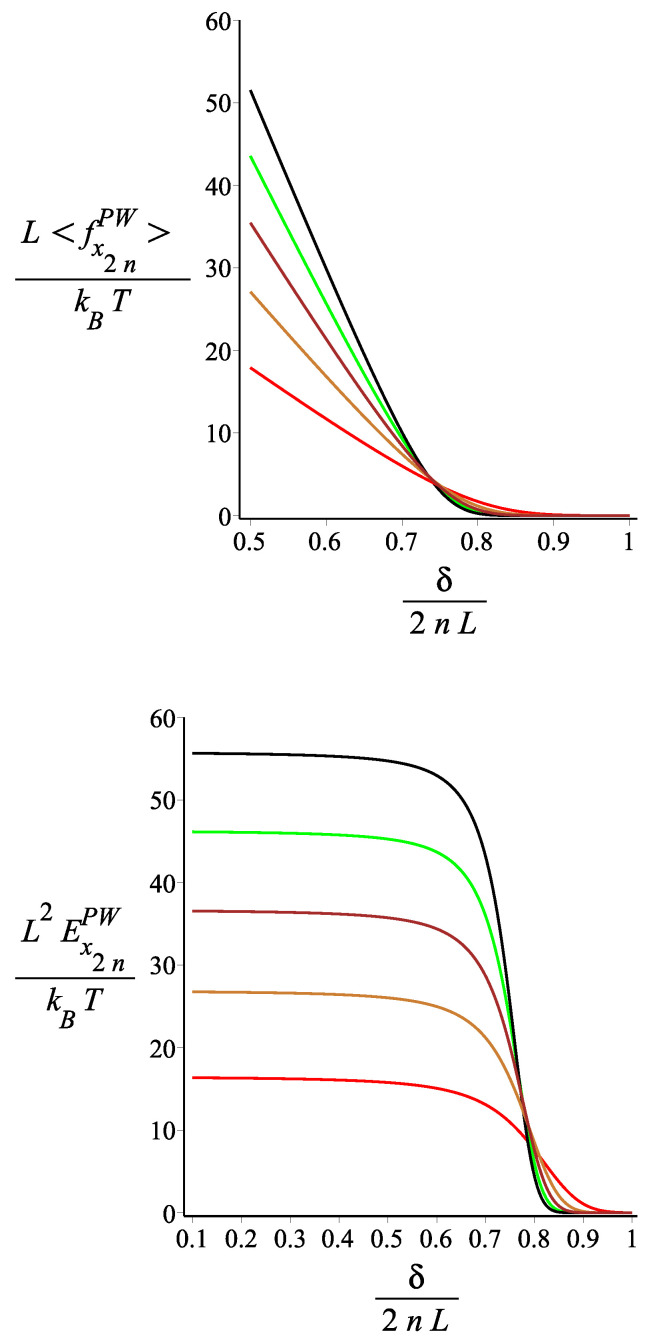
The **first panel**: The dimensionless force of the structure with an even number of arms exerted on the confining wall as a function of the dimensionless distance of the wall from the grafting point. The **second panel**: The dimensionless differential stiffness of the system with an even number of arms as a function of the dimensionless distance of the wall from the grafting point. The red, gold, brown, green, and black are associated with the fixed values of the single-arm bending stiffness parameter $\frac{l_{p}}{L}=12.5i$, where i=1,2,3,4,5, respectively. The other fixed parameters are: $\omega =\frac{\pi }{4}$, $\phi_{\mu }=0$, $\phi_{B}=0$n=2, and $K_{B}=3$.

**Figure 18:**
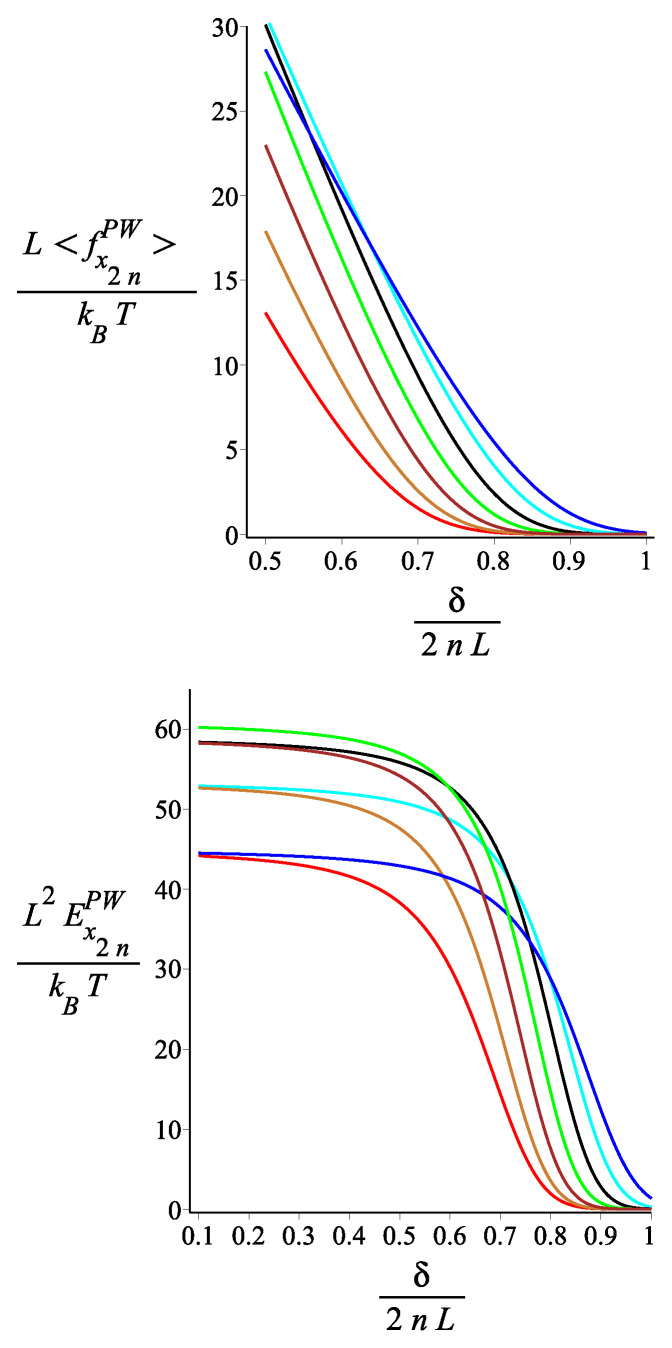
The **first panel**: The dimensionless force of the structure with an even number of arms exerted on the confining wall as a function of the dimensionless distance of the wall from the grafting point. The **second panel**: The dimensionless differential stiffness of the system with an even number of arms as a function of the dimensionless distance of the wall from the grafting point. The red, gold, brown, green, black, cyan, and blue are associated with the fixed values of the external field orientation parameter, $\phi_{B}=-\omega -\frac{\pi }{2}+\frac{\pi }{6}i$, where i=0,1,2,3,4,5,6, respectively. The other fixed parameters are: $\omega =\frac{\pi }{4}$, $\phi_{\mu }=0$, $\frac{l_{p}}{L}=30$, n=1, and $K_{B}=4$.

In Figure 16, Figure 17 and Figure 18, we show the force-extension relation and the corresponding differential stiffness for the confined spring for various values of the magnetic interaction ($K_{B}$), the single-arm bending stiffness ($l_{p}/L$), and the orientation of the external magnetic field ($\phi_{B}$). We point out that there is a finite force even for a distance δ for the grafting point much greater than the distance of the tip at T=0 and $K_{B}=0$ (for ω=π/4, and n=2, which would be 2L). The reason is that, because of the thermal fluctuations, the kinked structure unfolds. We see that by increasing $K_{B}$, the compressional force increases because the spring opens up and resists the confinement. In Figure 17, we see that, for small compression, the softer springs ($l_{P}/L$ small) exert the strongest force, but this dependence gets reversed as the compression increases. The reason is that for small compression, only the most flexible springs fluctuate strongly enough to reach out to such a long distance δ. As the compression increases, the bending energy becomes more important than the conformational entropy. It is interesting, as we see in Figure 18, how the orientation of the external field significantly affects the compressional response of the confined spring.

## 7. Conclusions

In this article, we investigated the conformations and the elasticity of a grafted semiflexible filament in two dimensions, with a regular alternating sequence of kinks and a magnetic moment at the free endpoint. Our results are all analytical. We assume that the weakly bending approximation (stiff limit) holds for each arm of the structure. However, we point out that this approximation is not very restrictive for the zigzag structure because the fluctuations add up with the number of arms, and the free tip can be strongly fluctuating with Gaussian-distributed fluctuations. In addition, if we consider the results concerning the orientational fluctuations of the free endpoint, these are exact (within the WLC model), irrespective of the stiffness of the arms. We calculated the response to a point force exerted at the tip and also to a rigid planar wall compressing the structure. Interestingly, the elastic response is strongly affected by all the parameters of the magnetic interaction (strength and orientation). This is due to the zigzag geometry and the orientational-positional coupling of the semiflexible arms. We point out that our force-extension relation for the compressional force on the confining wall can be extended in a straightforward way to a two-dimensional ((1+1)-dimensional) “brush” of grafted zigzag semiflexible springs with magnetic moment at the tip pushing against a confining wall. Of course, we have to assume that the grafting density is low in order to neglect the steric repulsion between different springs.

The behavior that we analyzed may prove useful in designing magneto-mechanical actuators susceptible to remote control of the semiflexible spring. Conversely, its sensitivity to the magnetic interaction may prove useful in measuring the external magnetic field or the attached magnetic moment.

## Data Availability

Not applicable.
